# Development and validation of a model for predicting in-hospital mortality in patients with sepsis-associated kidney injury receiving renal replacement therapy: a retrospective cohort study based on the MIMIC-IV database

**DOI:** 10.3389/fcimb.2024.1488505

**Published:** 2024-11-04

**Authors:** Caifeng Li, Ke Zhao, Qian Ren, Lin Chen, Ying Zhang, Guolin Wang, Keliang Xie

**Affiliations:** ^1^ Department of Critical Care Medicine, Tianjin Medical University General Hospital, Tianjin, China; ^2^ Department of General Surgery, Tianjin Medical University General Hospital, Tianjin, China; ^3^ Advertising Center, Tianjin Daily, Tianjin, China; ^4^ Department of Neurosurgery, Tianjin Medical University General Hospital Airport Hospital, Tianjin, China

**Keywords:** sepsis, acute kidney injury, renal replacement therapy, in-hospital mortality, predictive model, microbial infection

## Abstract

**Background:**

SAKI is a common and serious complication of sepsis, contributing significantly to high morbidity and mortality, especially in patients requiring RRT. Early identification of high-risk patients enables timely interventions and improvement in clinical outcomes. The objective of this study was to develop and validate a predictive model for in-hospital mortality in patients with SAKI receiving RRT.

**Methods:**

Patients with SAKI receiving RRT from the MIMIC-IV database were retrospectively enrolled and randomly assigned to either the training cohort or the testing cohort in a 7:3 ratio. LASSO regression and Boruta algorithm were utilized for feature selection. Subsequently, three machine learning models—CART, SVM and LR—were constructed, and their predictive efficacy was assessed using a comprehensive set of performance indicators. Feature importance analysis was performed to determine the contribution of each feature to a model’s predictions. Finally, DCA was employed to evaluate the clinical utility of the prediction models. Additionally, a clinical nomogram was developed to facilitate the interpretation and visualization of the LR model.

**Results:**

A total of 1663 adults were ultimately enrolled and randomly allocated into the training cohort (n = 1164) or the testing cohort (n = 499). Twenty-eight variables were evaluated for feature selection, with eight ultimately retained in the final model: age, MAP, RR, lactate, Cr, PT-INR, TBIL and CVP. The LR model demonstrated commendable performance, exhibiting robust discrimination in both the training cohort (AUROC: 0.73 (95% CI 0.70–0.76); AUPRC: 0.75 (95% CI 0.72–0.79); accuracy: 0.66 (95% CI 0.63–0.68)) and the testing cohort (AUROC: 0.72 (95% CI 0.68-0.76); AUPRC: 0.73 (95% CI 0.67–0.79); accuracy: 0.65 (95% CI 0.61–0.69)). Furthermore, there was good concordance between predicted and observed values in both the training cohort (χ2 = 4.41, p = 0.82) and the testing cohort (χ2 = 4.16, p = 0.84). The results of the DCA revealed that the LR model provided a greater net benefit compared to other prediction models.

**Conclusions:**

The LR model exhibited superior performance in predicting in-hospital mortality in patients with SAKI receiving RRT, suggesting its potential utility in identifying high-risk patients and guiding clinical decision-making.

## Introduction

Sepsis is a life-threatening condition characterized by multiple organ failure due to the host’s excess response to microbial infection, and is associated with high morbidity and mortality worldwide (Singer et al., 2016). The kidney is one of the most frequently affected organs during sepsis, resulting in the onset of sepsis-associated acute kidney injury (SAKI). Previous studies have shown that up to 50% of patients with sepsis suffer from acute kidney injury in intensive care units (ICU) (Peerapornratana et al., 2019; Poston and Koyner, 2019). The mortality rate in patients with SAKI is estimated to be 30%-45% (Poston and Koyner, 2019; See et al., 2019). Despite recent advances in the management of SAKI, in-hospital mortality remains notably high (Oh et al., 2012; Oh et al., 2014), especially in patients requiring renal replacement therapy (RRT) (Rosner, 2013). Therefore, accurate prognosis prediction is essential for disease understanding, patient-centered care, and shared decision-making.

Several novel biomarkers, such as tissue inhibitor of metalloproteinases-2 (TIMP-2), neutrophil gelatinase-associated lipocalin (NGAL), and insulin-like growth factor binding protein-7 (IGFBP-7), have been recognized as reliable indicators for early diagnosis, adverse outcomes and even mortality of SAKI. However, their sensitivity has yet to be validated in comprehensive multicenter studies (Bellomo et al., 2017). Many traditional scoring systems, including the Sequential Organ Failure Assessment (SOFA) score and the Simplified Acute Physiology Score II (SAPS-II), have been introduced to predict mortality in SAKI patients. Unfortunately, these scoring systems are unable to provide precise predictive estimates for specific disease processes and require laborious data collection and score calculation (Demirjian et al., 2011).

This study aims to develop a predictive model for in-hospital mortality in patients with SAKI receiving RRT, using readily available clinical information.

## Methods

### Study design

We conducted a retrospective observational study using data from the Marketplace for Medical Information in Intensive Care (MIMIC-IV) (Johnson et al., 2023), a publicly available database containing de-identified clinical data from patients admitted to the ICU of Beth Israel Deaconess Medical Center (BIDMC) in Boston, Massachusetts, USA, from 2008 to 2019.

As the MIMIC-IV database has obtained ethical approval from the Institutional Review Boards (IRBs) at BIDMC and MIT, additional ethical consent was waived for this study. Since all protected health information was de-identified, individual patient consent was not required. After completing the Collaborative Institutional Training Initiative (CITI) Examination, one author of our study (Caifeng Li) was granted access to the MIMIC-IV database (certification number: 33047414). This study was reported according to the Transparent Reporting of a multivariable prediction model for Individual Prognosis or Diagnosis (TRIPOD) statement (Collins et al., 2015).

Data extraction was performed using Structured Query Language (SQL), and the corresponding codes are openly available on GitHub (https://github.com/MIT-LCp/mimic-code). To facilitate the practical implementation and generalization of the predictive model, variables were collected based on the principles of early and easy acquisition.

### Study population

Patients with SAKI receiving RRT were identified retrospectively from the MIMIC-IV database based on the following inclusion criteria (Singer et al., 2016): adult patients who met the Sepsis 3.0 criteria, defined by a SOFA score ≥ 2 and had a suspected or confirmed infection (Singer et al., 2016) (Peerapornratana et al., 2019); patients who received RRT for AKI, defined as an increase in serum creatinine (Cr) by ≥ 0.3 mg/dL (26.5 µmol/L) within 48 hours, or an increase in Cr to ≥ 1.5 times of baseline within 7 days, or urine output < 0.5 mL/kg/h for 6 hours (Kellum and Lameire, 2013). When pre-admission Cr was not available, the first Cr measured on ICU admission was used as the baseline Cr for analysis (Angeli et al., 2015).

The exclusion criteria were as follows (1): patients aged < 18 years were excluded from the study (2); for patients with multiple ICU admissions, only the first admission record was included for analysis.

All eligible patients who met the inclusion and exclusion criteria were enrolled in the study and randomly assigned to either the training cohort for model development or the testing cohort for model evaluation.

### Data extraction

Data for each patient within the first 24 hours of ICU admission were extracted from the MIMIC-IV database using SQL. The following variables were collected (1): patient demographics, including age, gender, BMI and ethnicity (2); comorbidities, including heart failure, chronic pulmonary diseases, diabetes and cancer (3); vital signs, including heart rate (HR), mean arterial pressure (MAP), respiratory rate (RR) and temperature (4); severity scores, including SOFA score, Acute Physiology Score III (APS-III), Charlson comorbidity index and AKI-stage (5); laboratory and monitor parameters, including lactate, hemoglobin, platelet, WBC, Cr, calcium, sodium, potassium, prothrombin time-international normalized ratio (PT-INR), alanine aminotransferase, alkaline phosphatase, aspartate aminotransferase, total bilirubin (TBIL) and central venous pressure (CVP) (6); medications, including diuretics and vasopressors (7); in-hospital death record.

Missing values in exposure and risk factor variables were imputed using the mice algorithm. Subsequently, the model development and validation were performed on the imputed datasets using machine learning techniques.

### Feature selection

Feature selection was performed in the training cohort. We employed a rigorous feature selection approach to include the most relevant predictors for model construction, while avoiding any potential omissions. Therefore, we used the Boruta algorithm (Speiser et al., 2019) and the Least Absolute Shrinkage and Selection Operator (LASSO) algorithm (Vasquez et al., 2016), respectively, to obtain two sets of significant predictors. To ensure that only the most relevant and robust variables were included in the predictive model, we include the intersection of the two sets of predictors as the final features in the model. This strategy aimed at enhancing model accuracy and generalizability while minimizing the risk of overfitting or incorporating irrelevant predictors. As collinearity complicates the assessment of the unique contribution of each feature to the outcome, we employed a pairwise Pearson correlation matrix to evaluate the collinearity of clinical features, establishing a threshold of r > 0.8.

### Model development

The selected features were then used to build the model. Three machine learning classifiers—Classification and Regression Tree (CART), Support Vector Machine with Radial Kernel (SVM), and Logistic Regression (LR)—were employed for the development of predictive models. For consistency, each model included the same input features. Simultaneously, random hyperparameter searches were used to determine optimal hyperparameters for each model in the training cohort, using the area under the receiver operating characteristic curve (AUROC) as the optimization metric.

### Model validation

Model performance was estimated in the testing cohort. A variety of metrics were used to assess model performance, including the area under the precision-recall curve (AUPRC), AUROC, calibration curve, Brier score, and Hosmer-Lemeshow test. In addition, accuracy, sensitivity, specificity, positive predictive value (PPV), and negative predictive value (NPV) were also calculated to provide a comprehensive assessment. Furthermore, decision curve analysis (DCA) (Vickers et al., 2016) was performed to assess the models’ utility in decision-making by quantifying the net benefit at different threshold probabilities. Finally, feature importance analysis was performed to understand the contribution of each feature to model predictions, and a nomogram was developed to interpret and visualize the LR model.

### Comparison of the best model with traditional scoring systems

To determine whether the predictive model outperformed the traditional scoring system in predicting in-hospital mortality, we compared the best model with the traditional scoring system using the same dataset.

### Sample size calculation

To avoid overfitting and ensure accuracy, it is imperative to have a sufficient sample size when developing the predictive model. The sample size is calculated using the formula $n={\left(\frac{1.96}{\delta }\right)}^{2}$ ϕ(1−ϕ) *where* ϕ represents the expected outcome ratio ( ϕ = 0.46), δ is the set margin of error ( δ = 0.05) (Riley et al., 2020). According to this formula, the minimum sample size required for the model development process was 382 patients. The training cohort was adequate for model development. According to Collins’s recommendation for external validation of prognostic model, a minimum of 100 events is required, ideally 200 or more (Collins et al., 2016). The internal testing cohorts, with 230 events, met this criterion.

### Statistical analyses

The normality of data distribution was assessed using the Shapiro-Wilk test. Normally distributed continuous variables were reported as mean ± standard deviation (SD), while non-normally distributed continuous variables were reported as median (interquartile range, IQR). Continuous variables were analyzed using either the Student’s t-test or the Mann-Whitney U-test, depending on data distribution. Categorical variables were presented as absolute numbers (percentages) and compared using the Chi-square (χ2) test or the Fisher’s exact test, depending on the sample sizes. All data were analyzed using R software (version 4.0.3, R Foundation). A two-tailed p < 0.05 was considered statistically significant.

## Results

### Patient characteristics

**Figure 1:**
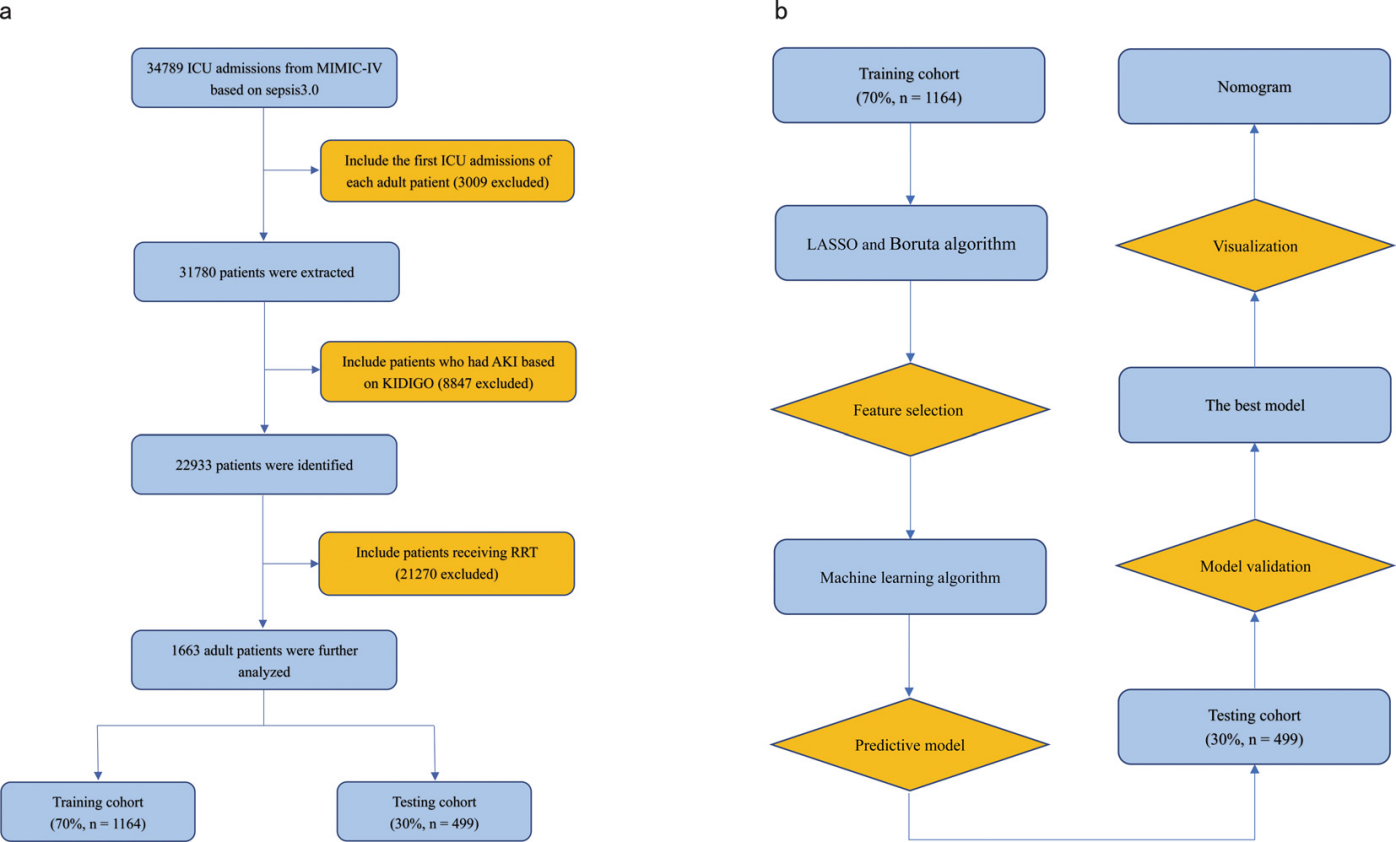
**(A)** Flowchart of patient selection. **(B)** Flowchart of model development and validation. ICU, Intensive Care Unit; RRT, Renal Replacement Therapy; KDIGO, Kidney Disease Improving Global Outcome; LASSO, Least Absolute Shrinkage and Selection Operator.

A total of 1663 patients were included in the final analysis according to predetermined inclusion and exclusion criteria. The process of patient recruitment and model development is illustrated in **Figure 1**, and the baseline characteristics are presented in **Table 1**. Preliminary statistical analysis revealed significant differences in several baseline characteristics between survivors and non-survivors. Compared with survivors, non-survivors were significantly older and showed statistical differences in ethnicity proportions. Regarding comorbidities, non-survivors exhibited a higher prevalence of cancer than survivors. Furthermore, non-survivors had higher BMI, HR, RR, APS-III, lactate, hemoglobin, WBC, PT-INR, TBIL and CVP, but lower MAP, temperature, platelet and Cr than survivors. Additionally, non-survivors exhibited a higher frequency of vasopressor use and a lower frequency of diuretic use.

**Table 1:** Patients’ baseline characteristics.

	Total	Training cohort	Testing cohort	P value	Survivor group	Non-survivor group	P value
N	1663	1164	499		904	759	
Age (year), (median [IQR])	65.29 [54.77, 74.78]	64.55 [54.61, 74.21]	66.15 [55.30, 76.23]	0.106	63.99 [53.51, 72.90]	66.82 [56.01, 76.32]	<0.001
Gender, n(%)				0.271			0.41
Female	658 (39.6)	450 (38.7)	208 (41.7)		349 (38.6)	309 (40.7)	
Male	1005 (60.4)	714 (61.3)	291 (58.3)		555 (61.4)	450 (59.3)	
BMI (kg/m2), (median [IQR])	29.62 [26.36, 34.66]	29.67 [26.26, 34.65]	29.57 [26.48, 34.60]	0.956	28.66 [26.02, 33.50]	30.37 [26.35, 35.56]	0.024
Ethnicity, n(%)				0.868			<0.001
Black	232 (14.0)	159 (13.7)	73 (14.6)		154 (17.0)	78 (10.3)	
White	975 (58.6)	684 (58.8)	291 (58.3)		546 (60.4)	429 (56.5)	
Others	456 (27.4)	321 (27.6)	135 (27.1)		204 (22.6)	252 (33.2)	
Comorbidity, n (%)							
Heart failure	775 (46.6)	548 (47.1)	227 (45.5)	0.588	429 (47.5)	346 (45.6)	0.477
Chronic pulmonary disease	483 (29.0)	347 (29.8)	136 (27.3)	0.32	271 (30.0)	212 (27.9)	0.389
Diabetes	438 (26.3)	294 (25.3)	144 (28.9)	0.142	243 (26.9)	195 (25.7)	0.622
Cancer	190 (11.4)	142 (12.2)	48 (9.6)	0.152	88 (9.7)	102 (13.4)	0.022
Baseline vital data							
Heart rate (bpm), (median [IQR])	89.84 [78.67, 103.71]	90.24 [79.24, 104.19]	89.18 [76.72, 102.65]	0.159	88.15 [76.67, 101.34]	92.07 [80.27, 105.81]	<0.001
Mean arterary pressure (mmHg), (median [IQR])	72.43 [66.64, 78.62]	72.46 [66.75, 78.68]	72.25 [66.36, 78.44]	0.395	73.36 [67.84, 79.81]	71.34 [65.47, 76.98]	<0.001
Respiratory rate (bpm), (median [IQR])	20.59 [17.77, 24.01]	20.78 [17.93, 24.12]	19.94 [17.34, 23.78]	0.026	19.60 [17.10, 23.12]	21.64 [18.72, 24.86]	<0.001
Temperature (°C), (median [IQR])	36.71 [36.41, 37.11]	36.74 [36.44, 37.12]	36.63 [36.36, 37.01]	0.002	36.75 [36.49, 37.12]	36.67 [36.31, 37.08]	0.001
Severity scores							
SOFA	5.00 [3.00, 7.00]	5.00 [3.00, 8.00]	5.00 [3.00, 7.00]	0.167	5.00 [3.00, 7.00]	5.00 [3.00, 8.00]	0.48
APS III	88.00 [68.00, 107.00]	89.00 [69.00, 109.00]	86.00 [67.00, 105.00]	0.051	80.00 [63.00, 100.00]	96.00 [78.00, 115.00]	<0.001
Charlson comorbidity index, (median [IQR])	7.00 [5.00, 9.00]	7.00 [5.00, 9.00]	7.00 [5.00, 9.00]	0.557	7.00 [5.00, 9.00]	7.00 [5.00, 9.00]	0.781
AKI-stage, n (%)				0.497			0.339
Stage 1	439 (26.4)	306 (26.3)	133 (26.7)		251 (27.8)	188 (24.8)	
Stage 2	278 (16.7)	187 (16.1)	91 (18.2)		152 (16.8)	126 (16.6)	
Stage 3	946 (56.9)	671 (57.6)	275 (55.1)		501 (55.4)	445 (58.6)	
Laboratory tests							
Lactate (mmol/L), (median [IQR])	3.50 [2.10, 6.10]	3.49 [2.07, 6.09]	3.50 [2.20, 6.17]	0.416	3.00 [1.80, 4.80]	4.32 [2.70, 7.55]	<0.001
Hemoglobin (*10^12^/L), (median [IQR])	10.50 [9.10, 12.10]	10.60 [9.20, 12.10]	10.40 [9.00, 12.10]	0.298	10.30 [9.00, 11.90]	10.78 [9.20, 12.40]	0.006
Platelet (*10^9^/L), (median [IQR])	127.00 [70.00, 196.00]	127.00 [69.00, 192.00]	129.00 [70.00, 203.50]	0.853	138.00 [81.75, 203.00]	119.00 [58.00, 190.00]	<0.001
WBC (*10^9^/L), (median [IQR])	15.00 [10.30, 21.00]	15.20 [10.40, 21.20]	14.10 [10.10, 20.60]	0.406	14.05 [9.80, 19.90]	16.10 [10.80, 22.70]	<0.001
Cr (mg/dL), (median [IQR])	3.40 [2.10, 5.10]	3.40 [2.10, 5.10]	3.30 [2.10, 5.10]	0.812	3.90 [2.40, 5.90]	2.90 [1.90, 4.30]	<0.001
Calcium (mg/dL), (median [IQR])	8.70 [8.10, 9.30]	8.60 [8.10, 9.30]	8.70 [8.20, 9.30]	0.062	8.64 [8.10, 9.30]	8.70 [8.15, 9.30]	0.242
Sodium (mmol/), (median [IQR])	135.00 [131.00, 139.00]	135.00 [131.00, 139.00]	136.00 [132.00, 139.00]	0.378	135.00 [132.00, 138.00]	135.00 [131.00, 139.00]	0.811
Potassium (mmol/L), (median [IQR])	4.10 [3.60, 4.60]	4.10 [3.60, 4.60]	4.00 [3.50, 4.50]	0.022	4.10 [3.60, 4.60]	4.10 [3.55, 4.60]	0.479
PT-INR, (median [IQR])	1.70 [1.30, 2.60]	1.70 [1.30, 2.60]	1.70 [1.30, 2.50]	0.348	1.60 [1.30, 2.20]	1.90 [1.40, 3.10]	<0.001
Alanine aminotransferase (IU/L), (median [IQR])	63.00 [23.00, 231.19]	59.73 [23.00, 232.00]	74.58 [25.00, 226.40]	0.099	63.00 [24.00, 228.45]	62.00 [23.00, 237.08]	0.918
Alkaline phosphatase (IU/L), (median [IQR])	120.47 [78.00, 173.80]	118.69 [76.00, 173.05]	127.00 [82.00, 174.54]	0.321	123.52 [82.00, 173.00]	116.00 [73.00, 174.82]	0.138
Aspartate aminotransferase (IU/L), (median [IQR])	134.00 [45.50, 456.81]	131.00 [44.00, 458.34]	144.84 [47.00, 440.00]	0.414	135.00 [45.00, 442.05]	132.00 [46.00, 468.49]	0.849
Total bilirubin (mg/dL), (median [IQR])	1.70 [0.70, 4.20]	1.80 [0.70, 4.30]	1.61 [0.60, 3.91]	0.098	1.55 [0.60, 3.80]	2.00 [0.80, 4.88]	<0.001
CVP	14.31 [12.37, 17.13]	14.33 [12.43, 17.16]	14.24 [12.23, 17.05]	0.354	13.75 [11.89, 15.75]	15.21 [13.19, 18.75]	<0.001
Medication, n (%)							
Diuretics	991 (59.6)	700 (60.1)	291 (58.3)	0.523	565 (62.5)	426 (56.1)	0.01
Vasopressors	995 (59.8)	702 (60.3)	293 (58.7)	0.581	481 (53.2)	514 (67.7)	<0.001

BMI, body mass index; IQR, interquartile range; APS, acute physiology score; SOFA, sequential organ failure assessment; AKI, acute kidney injury; WBC, white blood cells; Cr, Creatinine; PT-INR, prothrombin time-international normalized ratio.

Data were presented as mean standard deviation or median [interquartile range] or numbers (percentages).

### Feature selection

**Figure 2:**
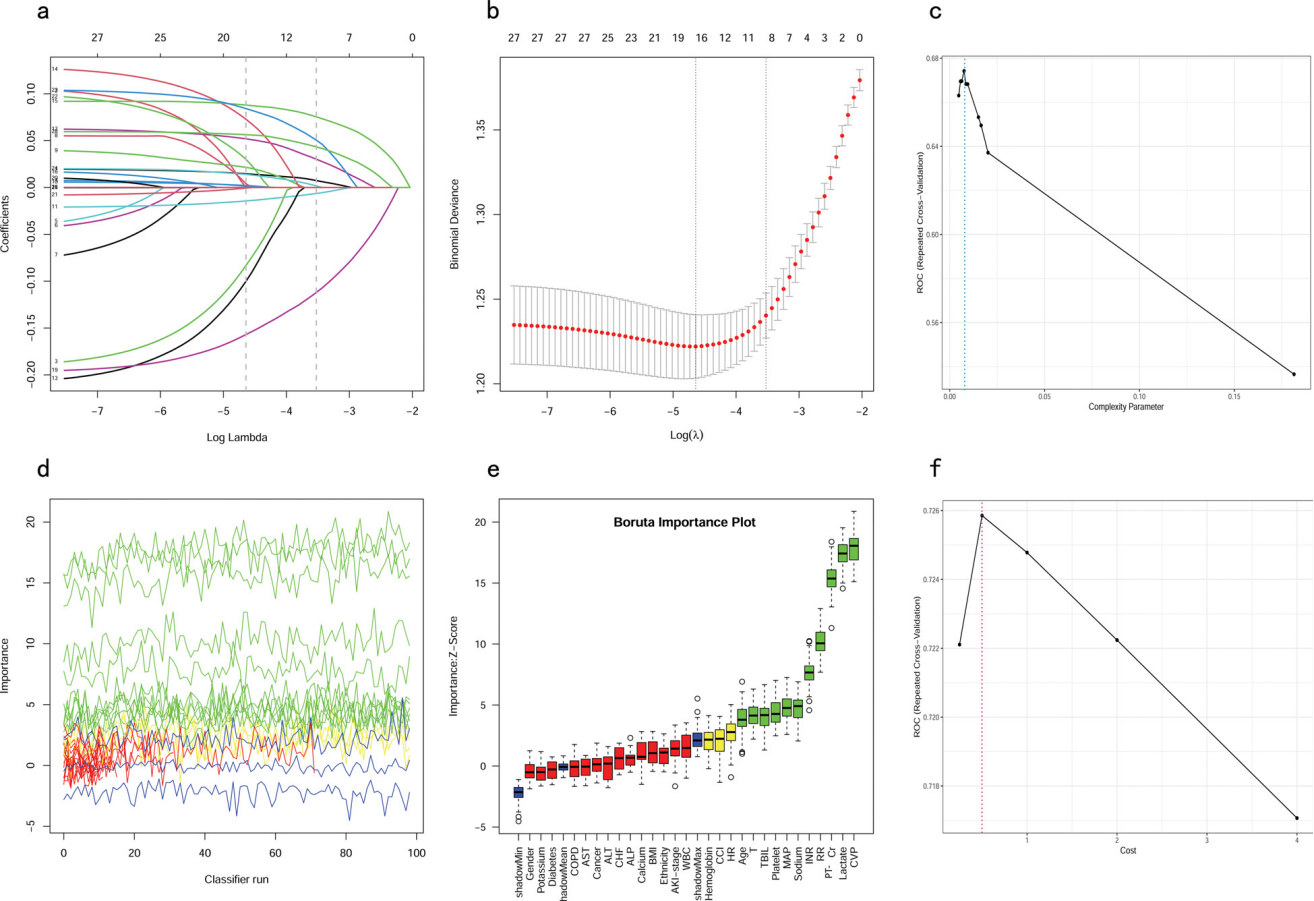
Feature selection and hyperparameter tuning. **(A, B)** Feature selection by using the Lasso regression. **(D, E)** Feature selection by using the Boruta algorithm. **(C)** Determination of optimal hyperparameters for the CART model. **(F)** Determination of optimal hyperparameters for the SVM model. LASSO, Least Absolute Shrinkage and Selection Operator; CART, Classification and Regression Tree; SVM, Support Vector Machine with Radial Kernel.

All patients in the training cohort were used for feature selection and model development. A total of 28 potential prognosis-related variables were screened using LASSO (**Figures 2A, B**) and Boruta (**Figures 2D, E**) algorithms, respectively. Finally, eight significant factors associated with in-hospital mortality were identified as independent predictors by both methods, including age, MAP, RR, lactate, Cr, PT-INR, TBIL and CVP. As shown in **Supplementary Figure S1**, none of the pairwise Pearson correlation values for these features exceeded 0.8, indicating the absence of collinearity. The regression coefficients for the variables in the LASSO regression are illustrated in **Supplementary Table S1**.

### Dose relationship between features and in-hospital mortality

Based on the outcomes of multivariate logistic regression, we further investigated the correlation between age, MAP, RR, lactate, Cr, PT-INR, TBIL and CVP and the in-hospital mortality. RCS is a conventional method for examining potential nonlinear relationships between the independent and dependent variables (Smith et al., 2016). Additionally, an akaike information criterion was employed to determine the optimal number of knots. We adjusted for confounding variables and performed a nonlinearity test before analyzing the dose-response relationship.

**Figure 3:**
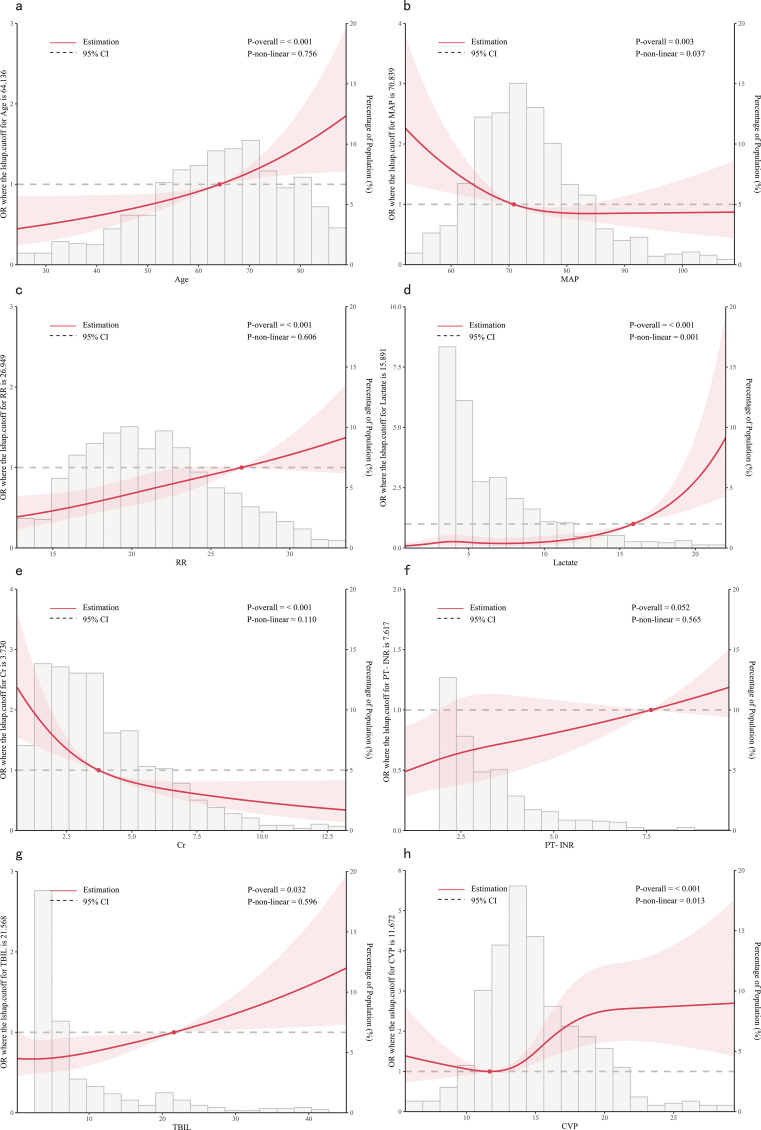
Dose-response relationships between features and in-hospital mortality. **(A)** Age; **(B)** MAP, Mean Arterial Pressure; **(C)** RR, Respiratory Rate; **(D)** Lactate; **(E)** Cr, creatinine; **(F)** PT-INR, Prothrombin Time-International Normalized Ratio; **(G)** TBIL, Total Bilirubin; **(H)** CVP, Central Venous Pressure.

From the dose-response plot (**Figure 3**), we observed a nonlinear correlation between MAP, lactate and CVP and in-hospital mortality (overall p < 0.05, nonlinear p < 0.05). It was noted that the risk of in-hospital mortality increased rapidly when lactate was higher than 15.89 mmol/L and MAP was lower than 70.84 mmHg. Regarding the J-shaped relation between CVP and in-hospital mortality, the plot showed a reduction of the risk within the lower range, which reached the lowest risk around 11.67 mmHg and then increased rapidly thereafter. However, the relationship between age, RR, Cr, TBIL, PT-INR and in-hospital mortality appeared to be linear (overall p < 0.05, nonlinear p > 0.05), with the risk threshold value being 64.14 years, 26.95 bpm, 3.73 mg/dL, 21.57 μmol/L and 7.62, respectively.

### Hyperparameter tuning

To ensure that each machine model achieved the best performance, we further optimized their hyperparameters. **Figures 2C, F**, illustrates the process of random hyperparameter searching for the CART model and the SVM model. The CART model was optimized with a cost complexity pruning (CP) value of 0.007561437. The optimal parameters for the SVM model were sigma = 0.1285088 and C = 0.5. As the LR model does not have hyperparameters, it was adjusted to the training data once, based on the stepwise procedure.

### Development of predictive models

**Figure 4:**
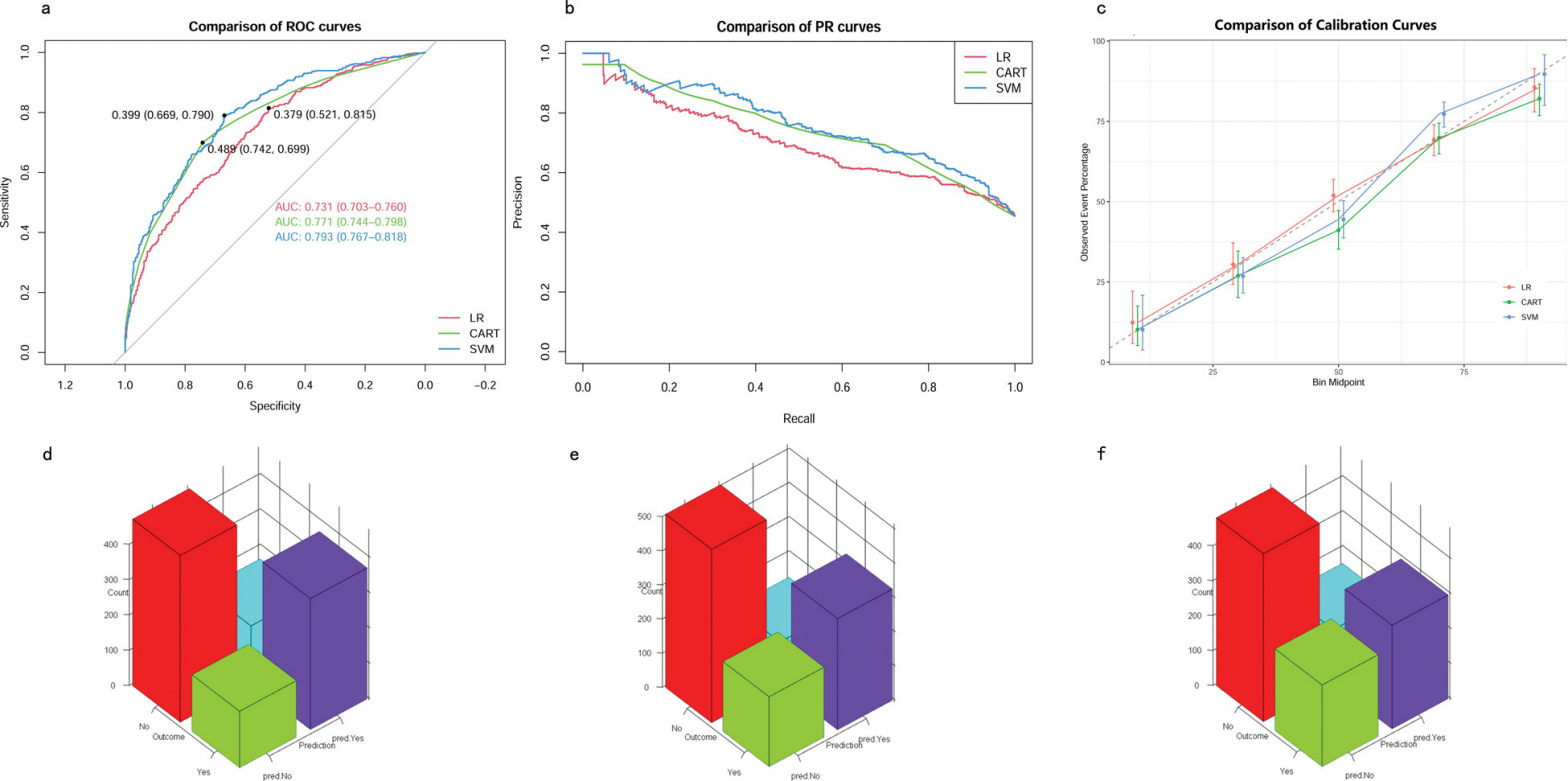
Model performance in training cohorts. **(A)** AUROCs. **(B)** AUPRCs. **(C)** Calibration plots. **(D–F)** Confusion matrix plots. AUROC, Area Under the Receiver Operating Characteristic curve; AUPRC, Area Under the Precision-Recall Curve; Classification and Regression Tree; SVM, Support Vector Machine with Radial Kernel; LR, Logistic Regression.

The eight aforementioned predictors, along with the optimal hyperparameters, were integrated into the predictive models. After 500 bootstrap resamples in the training cohort, the AUROCs for the CART model, SVM model, and LR model were 0.77 (95% confidence interval CI 0.74–0.80), 0.79 (95% CI 0.76–0.82) and 0.73 (95% CI 0.70–0.76), respectively (**Figure 4A**). The AUPRCs for the CART model, SVM model, and LR model were 0.77 (95% CI 0.70–0.82), 0.80 (95% CI 0.76–0.84) and 0.75 (95% CI 0.72–0.79), respectively (**Figure 4B**). In the CART model, SVM model, and LR model, the calibration curves demonstrated good concordance between predicted and observed outcomes (**Figure 4C**). The Brier scores for the CART model, SVM model, and LR model were 0.19 ± 0.20, 0.03 ± 0.02, and 0.21 ± 0.17, respectively. Hosmer-Lemeshow tests for the CART model, SVM model, and LR model were (χ2 = 9.95E-30, p = 1), (χ2 = 25.27, p = 0.001), and (χ2 = 4.41, p = 0.82), respectively. The accuracy for the CART model, SVM model, and LR model were 0.72 (95% CI 0.69–0.75), 0.71 (95% CI 0.68–0.73) and 0.66 (95% CI 0.63–0.68), respectively. The F1 scores for the CART model, SVM model, and LR model were 0.74 (95% CI 0.72–0.77), 0.75 (95% CI 0.72–0.78) and 0.71 (95% CI 0.68–0.74), respectively. Sensitivity, specificity, PPV, and NPV for the CART model were 0.74 (95% CI 0.71–0.77), 0.70 (95% CI 0.66–0.74), 0.75 (95% CI 0.71–0.78), and 0.69 (95% CI 0.65–0.73), respectively. The SVM model exhibited sensitivity, specificity, PPV, and NPV of 0.80 (95% CI 0.76–0.83), 0.61 (95% CI 0.57–0.66), 0.71 (95% CI 0.68–0.75), and 0.72 (95% CI 0.67–0.76). Sensitivity, specificity, PPV, and NPV for the LR model were 0.76 (95% CI 0.72–0.79), 0.56 (95% CI 0.52–0.60), 0.67 (95% CI 0.64–0.71), and 0.66 (95% CI 0.61–0.70). The SVM model demonstrated the best performance in the training cohort for predicting in-hospital mortality (**Figures 4D–F**).

### Validation of predictive models

**Figure 5:**
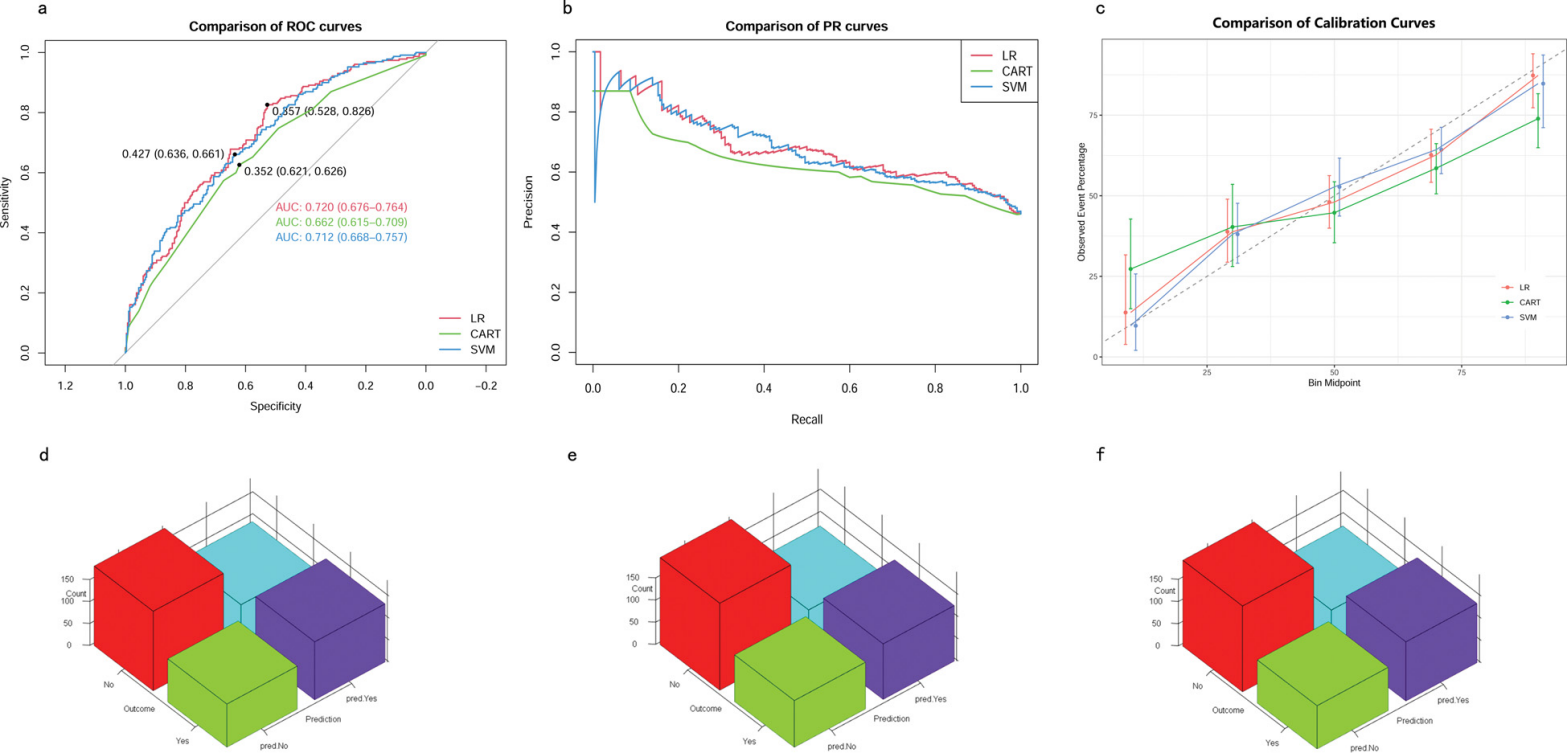
Model performance in testing cohorts. **(A)** AUROCs. **(B)** AUPRCs. **(C)** Calibration plots. **(D–F)** Confusion matrix plots. AUROC, Area Under the Receiver Operating Characteristic curve; AUPRC, Area Under the Precision-Recall Curve; CART, Classification and Regression Tree; SVM, Support Vector Machine with Radial Kernel; LR, Logistic Regression.

In the testing cohort, the AUROCs for the CART model, SVM model, and LR model were 0.66 (95% CI 0.62–0.71), 0.71 (95% CI 0.67–0.76), and 0.72 (95% CI 0.68–0.76), respectively (**Figure 5A**). The AUPRCs for the CART model, SVM model, and LR model were 0.56 (95% CI 0.43–0.60), 0.74 (95% CI 0.67–0.79), and 0.73 (95% CI 0.67–0.79), respectively (**Figure 5B**). Calibration curves (**Figure 5C**) and Hosmer-Lemeshow goodness-of-fit test demonstrated good concordance between predicted and observed outcomes in the SVM model (χ2 = 8.91, p = 0.35) and LR model (χ2 = 4.16, p = 0.84), but poor concordance in the CART model (χ2 = 37.44, p = 1.45E-06). The Brier scores for the CART model, SVM model, and LR model were 0.24 ± 0.22, 0.21 ± 0.18, and 0.21 ± 0.19, respectively. The accuracy for the CART model, SVM model, and LR model were 0.62 (95% CI 0.58–0.67), 0.64 (95% CI 0.59–0.68) and 0.65 (95% CI 0.61–0.69), respectively. The F1 scores for the CART model, SVM model, and LR model were 0.66 (95% CI 0.61–0.71), 0.69 (95% CI 0.64–0.73) and 0.69 (95% CI 0.64–0.73), respectively. Sensitivity, specificity, PPV, and NPV for the CART model were 0.67 (95% CI 0.62–0.73), 0.57 (95% CI 0.51–0.64), 0.65 (95% CI 0.59–0.71), and 0.60 (95% CI 0.53–0.67), respectively. The SVM model had sensitivity, specificity, PPV, and NPV of 0.73 (95% CI 0.68–0.78), 0.54 (95% CI 0.48–0.61), 0.65 (95% CI 0.60–0.71), and 0.63 (95% CI 0.56–0.70). Sensitivity, specificity, PPV, and NPV for the LR model were 0.72 (95% CI 0.66–0.77), 0.58 (95% CI 0.51–0.64), 0.67 (95% CI 0.61–0.73), and 0.64 (95% CI 0.57–0.70). As shown in (**Figures 5D–F**), the LR model exhibited superior performance to the other models, indicating good generalization and strong stability.

### Clinical utility of predictive models

**Figure 6:**
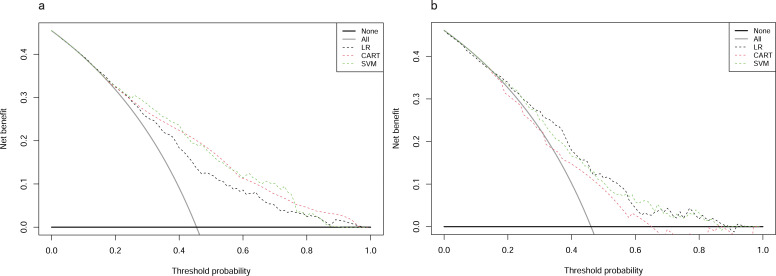
DCAs for models in training and testing cohorts. **(A)** Training cohort. **(B)** Testing cohort. DCA, Decision Curve Analysis; CART, Classification and Regression Tree; SVM, Support Vector Machine with Radial Kernel; LR, Logistic Regression.

In the training cohort, DCA revealed that when the threshold probability exceeded 20%, the mean net benefits of the CART model, SVM model and LR model for predicting in-hospital mortality were superior to the strategies of treating all or none of the patients (**Figure 6A**). This indicates that our predictive models, especially the LR model, provide significant clinical value by improving decision-making in identifying patients at higher risk for in-hospital mortality.

Likewise, in the testing cohort, the LR model demonstrated higher net benefits than the CART model and SVM model (**Figure 6B**). This highlights the robustness and reliability of the LR model, not only in the training cohort but also in external validation settings, reinforcing its potential as a practical tool for clinical decision-making.

### Feature importance of predictive models

**Figure 7:**
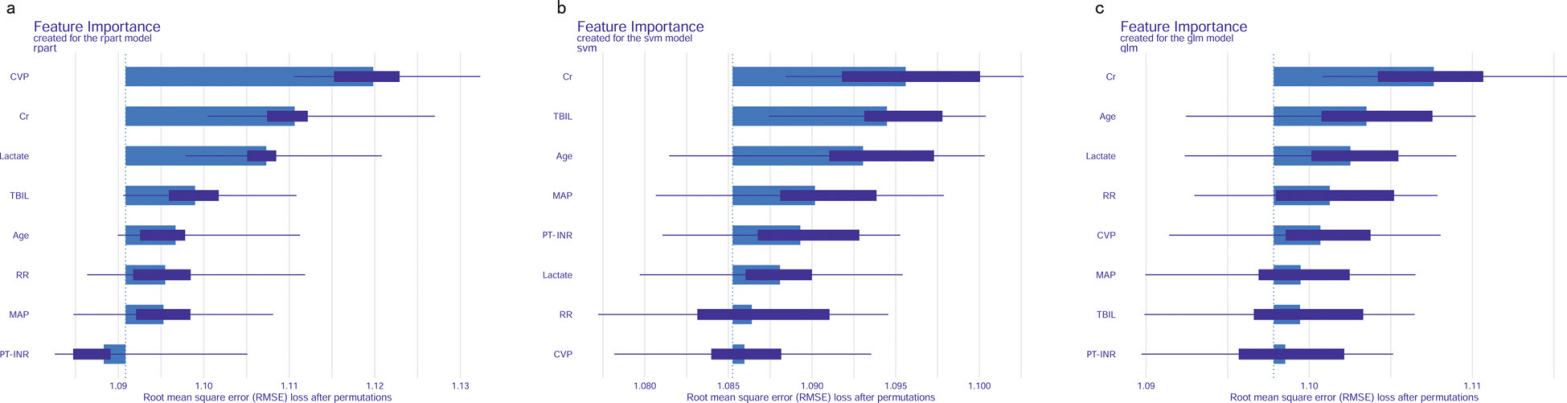
Feature importance of predictive models. **(A)** CART model. **(B)** SVM model. **(C)** LR model. MAP, Mean Arterial Pressure; RR, Respiratory Rate; PT-INR, Prothrombin Time-International Normalized Ratio; TBIL, Total Bilirubin; CVP, Central Venous Pressure; Cr, creatinine.

Permutation feature importance analysis revealed the key predictors for prediction. The results varied across the three models. In the CART model, CVP was the most influential factor, followed by Cr, lactate, TBIL, age, RR, MAP, and PT-INR (**Figure 7A**). In the SVM model, Cr was the top predictor, with TBIL, age, MAP, INR, lactate, RR, and CVP coming next (**Figure 7B**). In the LR model, Cr emerged as the most crucial predictor, followed by age, lactate, RR, CVP, MAP, TBIL, and PT-INR (**Figure 7C**). To synthesize the significance of these features across all three models, we introduced the concept of a rank score. The most important feature in each model received a full score of 8, decreasing sequentially to 1 for the least significant feature. Cr achieved a rank score of 23, ranking it at the top. This underscores Cr’s substantial predictive value for predicting in-hospital mortality risk in SAKI patients receiving RRT. Other top features were age, lactate, TBIL, CVP, RR, MAP and PT-INR, with a rank score of 17, 15, 14, 13, 10, 10 and 6, highlighting their importance in predictive models for this clinical scenario.

### The best model and its explainability

By comparing the model’s performance across the training cohort, testing cohort and clinical usage, we concluded that the LR model exhibited superior total performance but minimal overfitting. The advantages of the LR model also lie in its simplicity, interpretability, and no need for tuning. The result of the logistic regression analysis is showed in **Supplementary Figure S2**.

**Figure 8:**
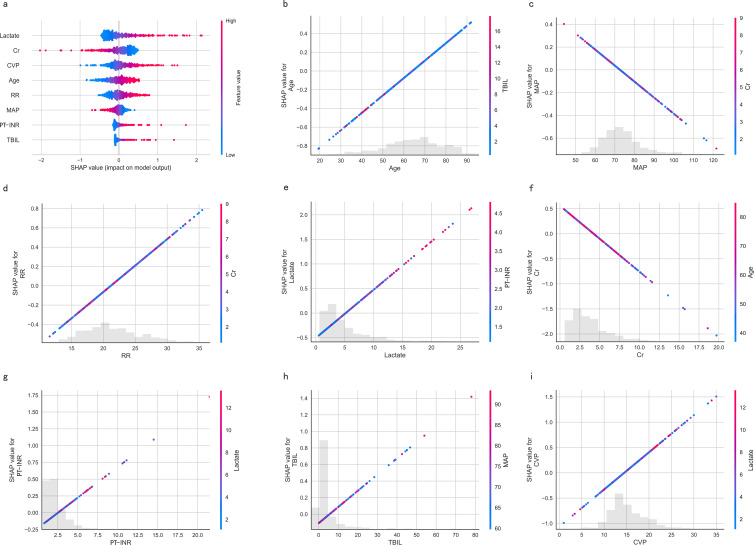
SHAP-based interpretation for the LR model. **(A)** The Beeswarm plot depicts the influence of the eight features across all samples. Combining feature importance and feature effect, Beeswarm ranks the features according to the sum of the SHAP across all samples (y-axis). One row in the plot represents one feature, and each dot represents the feature Shapley value for one sample; colors represent feature values (red for high, blue for low). The x-axis represents the influence on the model’s output, with positive values increasing risk and negative values decreasing risk. **(B-I)** SHAP dependence plots show the effect of a single feature across the whole dataset. MAP, Mean Arterial Pressure; RR, Respiratory Rate; PT-INR, Prothrombin Time-International Normalized Ratio; TBIL, Total Bilirubin; CVP, Central Venous Pressure; Cr, creatinine.

**Figure 9:**
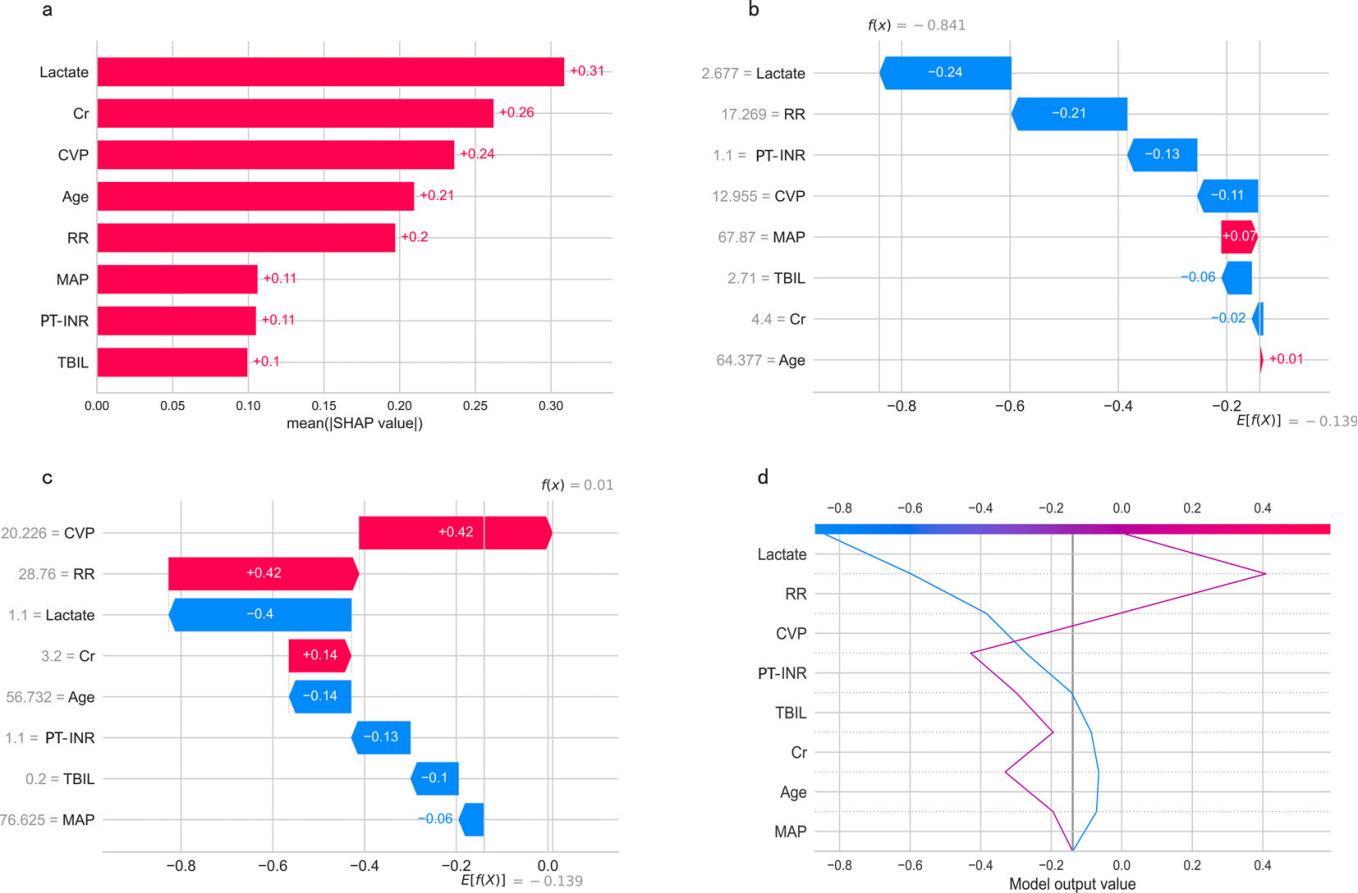
**(A)** Feature ranking according to the mean absolute Shapley values. **(B, C)** The SHAP waterfall plots for explanations of individual predictions. **(D)** The SHAP decision plot shows how the LR model arrives at its prediction.SHAP, SHapley Additive exPlanations; MAP, Mean Arterial Pressure; RR, Respiratory Rate; PT-INR, Prothrombin Time-International Normalized Ratio; TBIL, Total Bilirubin; CVP, Central Venous Pressure; Cr, creatinine.

The SHapley Additive exPlanations (SHAP) summary plot (**Figure 8A**) and dependence plot (**Figures 8B–I**) illustrate the contributions of the eight predictors within the LR model. SHAP values above zero indicate an increased risk of in-hospital mortality, whereas values below zero suggest a decreased risk. For instance, lower lactate (blue) generally yields SHAP values below zero, indicating a reduced risk of in-hospital mortality in patients with low lactate. Moreover, **Figure 9A** portrays the feature rankings based on the average absolute SHAP value in the LR model. Lactate, Cr, and CVP emerged as the three most influential features in predictive power. Higher lactate levels, Lower Cr levels, and increased CVP indicated a greater likelihood of death onset. Then, the SHAP waterfall plot explains the effect each feature on individual predictions in the LR model. The SHAP waterfall plots display explanations for individual patients, with the weight of each feature represented in either blue or red depending on whether it favors the outcome or not. In case 1, the LR model showed a probability of death (69.88%), which may be attributed to the likelihood that an elder patient with lower MAP possesses a greater probability of death, despite the presence of favorable indicators, such as a relatively normal lactate, RR, INR, and CVP (**Figure 9B**). In Case 2, the LR model forecasted a mortality probability of 50.25%. The interpreter algorithm discerned that a patient with increased CVP, elevated RR, and higher Cr might be predisposed to an unfavorable outcome, despite the presence of negative prognostic factors, such as lower INR, lactate, and a younger age, as well as normal MAP and TBIL (**Figure 9C**). The SHAP decision plot (**Figure 9D**) shows how features influence the models’ decision-making for individual samples.

### Visualization of the best model

**Figure 10:**
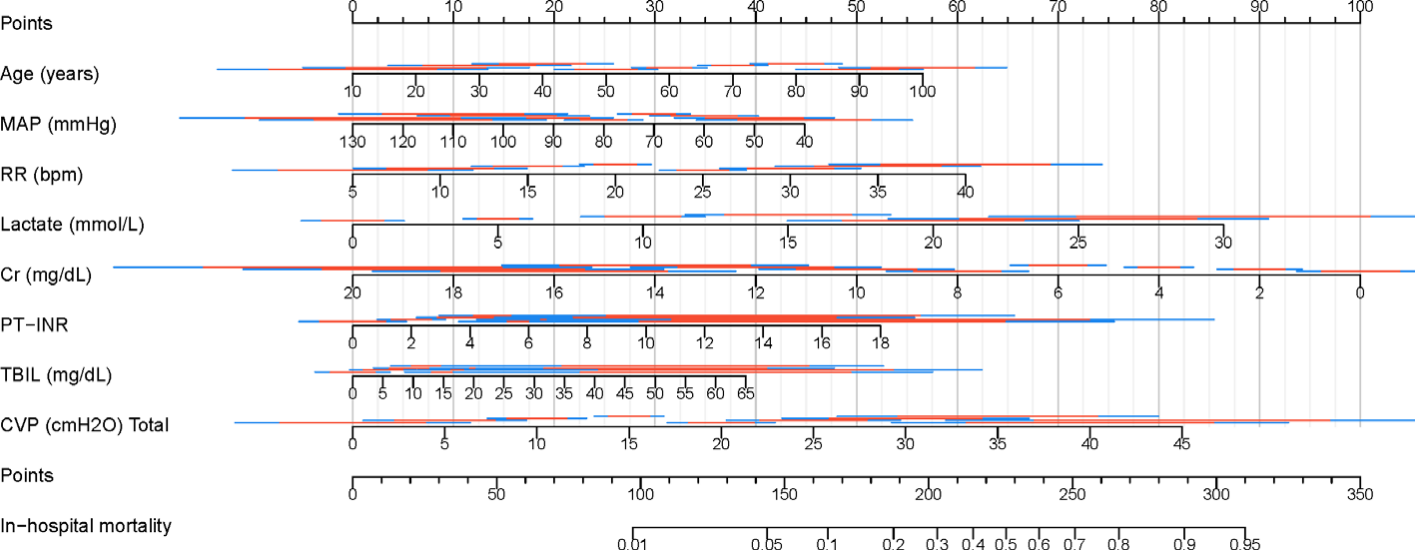
Nomogram for predicting in-hospital mortality in patients with SAKI receiving RRT. MAP, Mean Arterial Pressure; RR, Respiratory Rate; PT-INR, Prothrombin Time-International Normalized Ratio; TBIL, Total Bilirubin; CVP, Central Venous Pressure; Cr, creatinine.

Finally, a clinical nomogram was constructed to interpret and visualize the LR model (**Figure 10**). By assigning a weighted point to each independent risk factor on the point scale, the total point for each patient can be calculated, and the corresponding probability of in-hospital mortality can be determined by drawing a vertical line from the Total Points value to the Risk axis. A higher total point of all risk factors refers to a higher in-hospital mortality rate.

### Comparing the best model with traditional scoring systems

**Figure 11:**
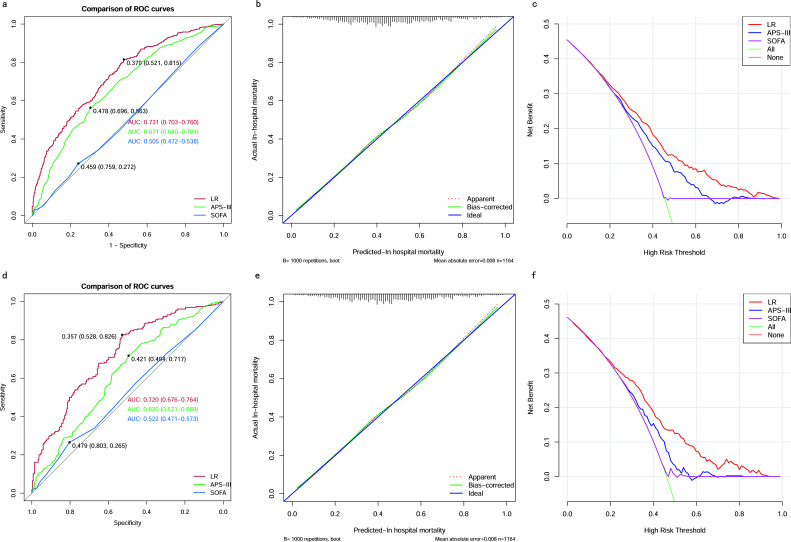
Comparison between the LR model and Traditional Scoring Systems. **(A)** AUROCs in training cohort. **(B)** Calibration plot for LR model in training cohort. **(C)** DCAs in training cohort. **(D)** AUROCs in testing cohort. **(E)** Calibration plot for LR model in testing cohort. **(F)** DCAs in testing cohort. AUROC, Area Under the Receiver Operating Characteristic curve; DCA, Decision Curve Analysis; SOFA, sequential organ failure assessment; APS-III, acute physiology scores III; LR, Logistic Regression.

We also compared the performance of the LR model with traditional scoring systems. AUROC of the LR model in the training and testing cohort was 0.73 (95% CI 0.70–0.76) and 0.72 (95% CI 0.68-0.76), which significantly outperformed the SOFA score (training cohort: 0.51 (95% CI 0.47-0.54); testing cohort: 0.52 (95% CI 0.47-0.57)), and the APS-III (training cohort: 0.67 (95% CI 0.64-0.70); testing cohort: 0.62 (95% CI 0.57-0.67)). The LR model exhibited superior discrimination in predicting in-hospital mortality in patients with SAKI receiving RRT when compared with the SOFA score, and the APS-III (**Figures 11A, D**). The calibration curves (**Figures 11B, E**) showed that the apparent line and the bias-corrected line deviated slightly from the ideal line, indicating good concordance between the predictions and observations in both the training and testing cohort. DCA indicated that the LR model could provide a superior net clinical benefit over previously reported scoring systems. As illustrated in **Figures 11C, F**, the LR model-directed medical intervention could provide higher net benefits than other scoring systems when the probability threshold (PT) exceeded 0.2.

## Discussion

Mortality prediction for ICU patients is crucial for improvement of outcomes and efficient utilization of resources. The SOFA score, APS-III and AKI-stage are the most commonly used ICU severity scores for predicting short-term mortality in patients with sepsis (Lambden et al., 2019; Hu et al., 2021). However, research showed that the performance of these non-specific scoring systems was disappointing in patients with SAKI (Le Gall et al., 1993). OHNUMA T et al. also examined and compared the performance of traditional scoring systems among patients with SAKI requiring RRT, but none of them achieved an AUC greater than 0.70 (Ohnuma et al., 2015).

A simplified and applicable risk model represents a practical tool that can be easily used in the early stages. Benefiting from the 10-year data collection of the MIMIC-IV database and the rapid development of machine learning algorithms, we have developed the LR model and constructed a simple nomogram based on eight easily available features. Elderly patients were more likely to develop AKI and experience poorer prognoses following an AKI episode, as demonstrated in numerous clinical studies (Ishani et al., 2009; Chao et al., 2015; Rhee et al., 2016; Fabbian et al., 2019). Given its simplicity and ease of observation, the respiratory rate usually serves as a valuable indicator for identifying high-risk patients (Wang et al., 2021). According to classical theories, hypotension and associated ischemia were considered the primary lesion in SAKI (Poston and Koyner, 2019). Another study also demonstrated that low blood pressure during AKI may contribute to increased mortality (Khalil et al., 2018). CVP was identified as an independent predictor of short-term mortality in critically ill patients with AKI, with this effect being more pronounced in those with severe AKI (Huang et al., 2021). Therefore, intensivists must maintain appropriate blood pressure and volume status to mitigate the complications and mortality associated with AKI in older patients (Darden et al., 2021). Lactate, a marker that reflects arterial perfusion and oxygen supply, has been demonstrated to independently predict mortality in patients with SAKI (Shen et al., 2022). Both lactate clearance and lactate levels after 24 hours of continuous renal replacement therapy (CRRT) were found to be independently associated with mortality in patients with SAKI undergoing CRRT (Passos et al., 2016). Serum Cr is a commonly used marker for the assessment of renal function in severity scores, such as SOFA and APACHE-II scores. However, changes in serum Cr are delayed due to renal reserve and the kinetics of AKI (Poston and Koyner, 2019). Our study showed that higher Cr levels exhibited a protective effect for SAKI, consistent with the findings of Cerdá et al (Cerdá et al., 2007) and da Hora Passos et al (da Hora Passos et al., 2017). in critically ill patients with AKI requiring CRRT. This can be attributed to the fact that severe AKI can lead to oliguria and fluid accumulation, which in turn dilute Cr concentrations and thus underestimate the severity of AKI (Macedo et al., 2010). In previous research, the MELD score and its components, including TBIL and PT-INR, were found to be associated with AKI development following liver transplantation, indicating TBIL and PT-INR were independent predictive predictors for AKI (Guo et al., 2020).

We compared the performance of our LR model with traditional scoring systems, specifically the SOFA and APS-III scores, for predicting in-hospital mortality in patients with SAKI undergoing RRT. Results showed that the LR model significantly outperformed traditional scoring systems in both the training and test cohorts. The AUROC for the LR model was 0.73 (95% CI 0.70-0.76) in the training cohort and 0.72 (95% CI 0.68-0.76) in the testing cohort, which was significantly higher than the SOFA score (training cohort: 0.51; testing cohort: 0.52) and the APS-III score (training cohort: 0.67; testing cohort: 0.62). The superior discrimination of the LR model highlights its ability to predict mortality risk more accurately than the traditional systems, particularly the SOFA score, which showed poor discriminatory power. This improved performance can be attributed to the inclusion of more relevant and comprehensive variables in the LR model, which better captures the complexity of the SAKI patient’s condition. The calibration curves further supported the robustness of the LR model, showing good agreement between predicted and observed outcomes in both the training and test cohorts, despite minor deviations from the ideal line. This suggests that our model not only predicts well, but also maintains consistency across different data sets. In addition, DCA indicated that the LR model provided superior net clinical benefit over traditional scoring systems, especially when the probability threshold exceeded 0.2. This means that when the predicted probability of mortality exceeds 20%, the LR model provides greater clinical utility and guides medical intervention more effectively than the SOFA or APS-III scores. This reinforces the clinical relevance of the LR model, as it enables more accurate risk stratification and informed decision-making, potentially leading to better patient outcomes in this high-risk population.

The potential strength of the LR model lies in its simplicity, which significantly reduces the computational time while maintaining high accuracy. This model is based on eight easily available variables in clinical practice, allowing for rapid stratification of patients into different severity levels with different mortality rates. The LR model exhibited robust discrimination and satisfactory calibration in the training cohort and the testing cohort. Both the Hosmer-Lemeshow test and calibration curve confirmed the excellent calibration of our model. In addition, DCA curves demonstrated that the nomogram yielded greater net benefits across a broad range of threshold probabilities in the training and testing cohort.

The LR model is helpful in multiple ways. First, it serves as a valuable tool for clinicians to identify high-risk patients with SAKI undergoing RRT. By accurately identifying those at greater risk of adverse outcomes, the model enables healthcare providers to implement targeted interventions tailored to the specific needs of these patients. This approach not only promotes personalized medicine, but also increases the overall effectiveness of treatment strategies, leading to improved patient outcomes. Second, the LR model empowers patients and their families by providing clear and accurate predictive information essential for informed decision making. By understanding potential risks and expected outcomes, patients and their families can become more actively involved in their care, fostering a collaborative relationship with healthcare providers. This transparency not only helps reduce anxiety and uncertainty, but also empowers families to make decisions that align with their values and preferences. Overall, the LR model plays a critical role in improving both clinical decision-making and patient engagement, ultimately contributing to a higher standard of care in the management of SAKI patients.

Despite the strengths of this study, several limitations must be acknowledged. First, the retrospective observational design inherently introduces the possibility of selection bias and the presence of unknown confounders, which cannot be completely eliminated. To mitigate this, we implemented strict inclusion and exclusion criteria to ensure that only representative cases were analyzed. Second, although there were some missing values in the dataset, we used multiple imputation methods to address these gaps, striving to produce unbiased estimates that more accurately reflect the true values. In addition, we must consider the potential influence of feature selection methods, which can lead to overfitting and thus affect the generalizability of the model. Finally, although we have conducted a thorough internal validation of the model’s performance, it is imperative to pursue external validation using diverse data sources. Future steps include plans for prospective validation in clinical settings, which will further enhance the robustness and applicability of our findings.

## Conclusions

By combining eight risk factors, we developed a simplified LR model to predict in-hospital mortality in patients with SAKI receiving RRT with satisfactory performance. Nonetheless, external validation using a new cohort is necessary for future research.

## Data Availability

Publicly available datasets were analyzed in this study. This data can be found here: The datasets are available on the website of PhysioNet (MIMIC-IV: https://physionet.org/content/mimiciv/1.0/).

## Supplementary material

Supplementary Figure 1 The Spearman or Pearson correlation matrix of continuous features. MAP, Mean Arterial Pressure; RR, Respiratory Rate; PT-INR, Prothrombin Time-International Normalized Ratio; TBIL, Total Bilirubin; CVP, Central Venous Pressure; Cr, creatinine.

Supplementary Figure 2 The result of the logistic regression analysis.

## Glossary

RRT: Renal Replacement Therapy
AKI: Acute Kidney Injury
MIMIC-IV: Medical Information Mart for Intensive Care IV
OR: Odds Ratio
SAKI: Sepsis-induced Acute Kidney Injury
ICU: Intensive Care Unit
LASSO: Least Absolute Shrinkage and Selection Operator
AUC: Area Under the Curve
DCA: Decision Curve Analysis
MAP: Mean Arterial Pressure
PT-INR: Prothrombin Time-International Normalized Ratio
TBIL: Total Bilirubin
CVP: Central Venous Pressure
NGAL: Neutrophil Gelatinase-Associated Lipocalin
IGFBP-7: Insulin-like Growth Factor Binding Protein-7
TIMP-2: Tissue Inhibitor of Metalloproteinases-2
BIDMC: Beth Israel Deaconess Medical Center
IRBs: Institutional Review Boards
CITI: Collaborative Institutional Training Initiative
SOFA: Sequential Organ Failure Assessment
KDIGO: Kidney Disease Improving Global Outcome
IQR: Interquartile Range
SMD: Standardized Mean Difference
SD: Standard Deviation
EGDT: Early Goal-Directed Therapy
CKD: Chronic Kidney Disease
BMI: Body Mass Index
Cr: Creatinine
SQL: Structured Query Language
APS: Acute Physiology Score
SAPS: Simplified Acute Physiology Score
WBC: White Blood Cells
PT: Probability Threshold
MELD: Model for End-Stage Liver Disease
RR: Respiratory Rate
HR: Heart Rate
LR: Logistic Regression
CART: Classification and Regression Tree
SVM: Support Vector Machine with Radial Kernel
AUROC: Area Under the Receiver Operating Characteristic curve
AUPRC: Area Under the Precision-Recall Curve
PPV: Positive Predictive Value
NPV: Negative Predictive Value
CP: Complexity Pruning
CI: Confidence Interval
SHAP: SHapley Additive exPlanations
