# Supplementary figures and images for: Development and validation of a model for predicting in-hospital mortality in patients with sepsis-associated kidney injury receiving renal replacement therapy: a retrospective cohort study based on the MIMIC-IV database
Source: Front Cell Infect Microbiol. 2024 Nov 4;14:1488505. doi: 10.3389/fcimb.2024.1488505 (PMC11570588; doi:10.3389/fcimb.2024.1488505)

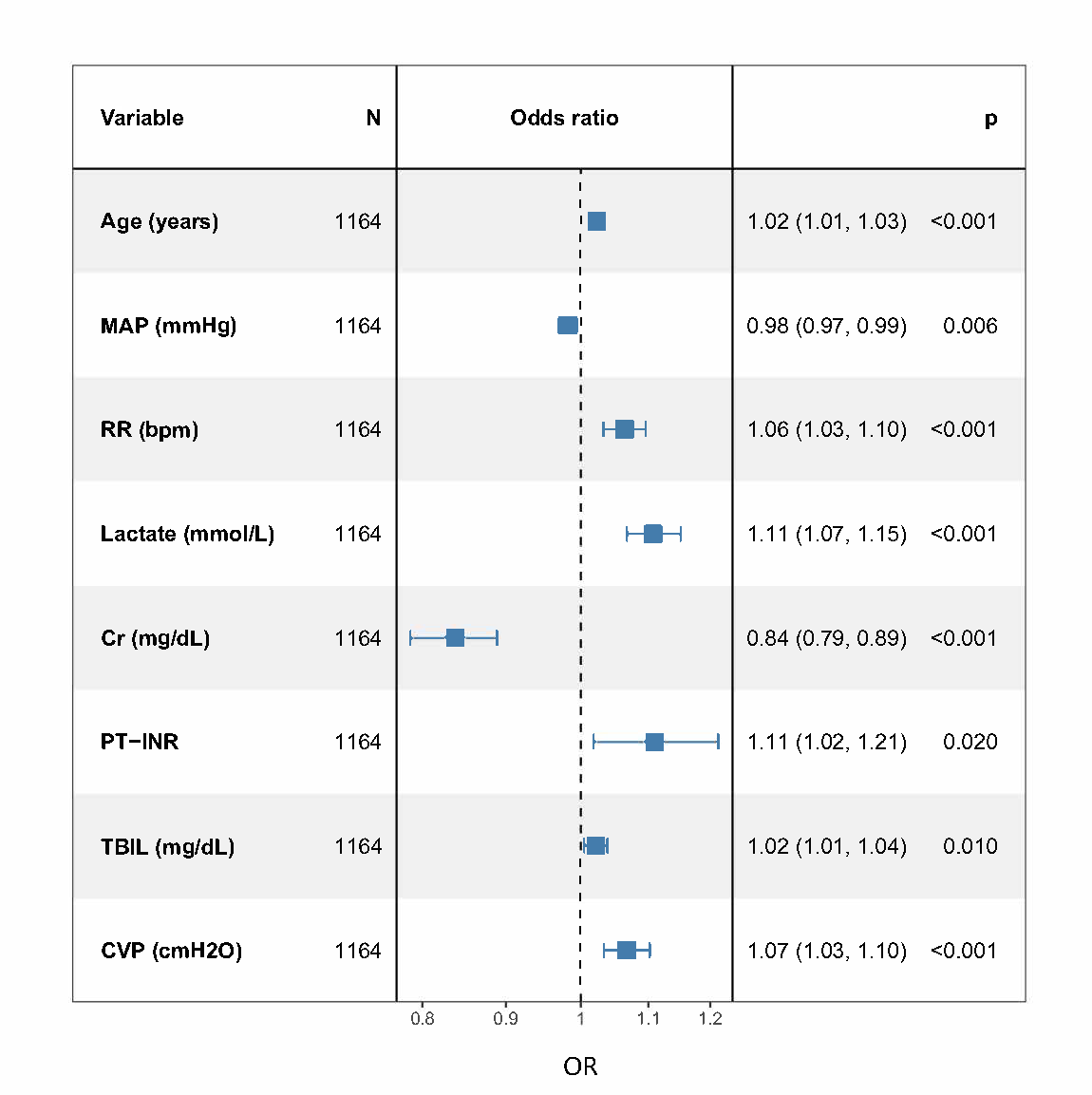

Supplement: Supplementary Figure 2 — The result of the logistic regression analysis. [file Image2.tif]
