# Supplementary material for: Development and validation of a model for predicting in-hospital mortality in patients with sepsis-associated kidney injury receiving renal replacement therapy: a retrospective cohort study based on the MIMIC-IV database
Source: Front Cell Infect Microbiol. 2024 Nov 4;14:1488505. doi: 10.3389/fcimb.2024.1488505 (PMC11570588; doi:10.3389/fcimb.2024.1488505)
Supplement: Supplementary file 3 [file Table1.docx]

**Table S1** The results of the LASSO regression analysis.

| Variables | coefficients |
| --- | --- |
| Age | 0.007413 |
| MAP | -0.0064 |
| RR | 0.03026 |
| Lactate | 0.075374 |
| Cr | -0.11206 |
| PT-INR | 0.0505 |
| TBIL | 0.001732 |
| CVP | 0.04338 |

MAP: Mean Arterial Pressure; RR: Respiratory Rate; Cr: Creatinine; PT-INR: Prothrombin Time-International Normalized Ratio; TBIL: Total Bilirubin; CVP: Central Venous Pressure.
