# Supplementary material for: Development and validation of a model for predicting in-hospital mortality in patients with sepsis-associated kidney injury receiving renal replacement therapy: a retrospective cohort study based on the MIMIC-IV database
Source: Front Cell Infect Microbiol. 2024 Nov 4;14:1488505. doi: 10.3389/fcimb.2024.1488505 (PMC11570588; doi:10.3389/fcimb.2024.1488505)
Supplement: Supplementary file 4 [file Table2.docx]

**Table S2** The result of the logistic regression analysis.

| **Characteristic** | **OR** | **95% CI** | **p-value** |
| --- | --- | --- | --- |
| Age | 1.02 | 1.01, 1.03 | <0.001 |
| MAP | 0.98 | 0.97, 1.0 | 0.006 |
| RR | 1.06 | 1.03, 1.10 | <0.001 |
| Lactate | 1.11 | 1.07, 1.15 | <0.001 |
| Cr | 0.84 | 0.79, 0.89 | <0.001 |
| PT-INR | 1.11 | 1.02, 1.21 | 0.020 |
| TBIL | 1.02 | 1.01, 1.04 | 0.010 |
| CVP | 1.07 | 1.03, 1.10 | <0.001 |
| OR: Odds Ratio; CI: Confidence Interval | | | |
